# Self-Efficacy and Acceptance of Illness Among Older Patients with Heart Failure

**DOI:** 10.3390/bs15050679

**Published:** 2025-05-15

**Authors:** Urszula Religioni, Małgorzata Kupisz-Urbańska, Wiktoria Niegowska, Agnieszka Drab, Piotr Czapski, Katarzyna Januszewska-Mukarker, Jarosław Pinkas, Beata Gellert, Janusz Ostrowski, Piotr Jankowski

**Affiliations:** 1 School of Public Health, Centre of Postgraduate Medical Education, 01-826 Warsaw, Poland; 2 Department of Geriatrics, Medical Centre of Postgraduate Education, 00-416 Warsaw, Poland; 3 Department of Internal Medicine and Geriatric Cardiology, Medical Centre of Postgraduate Education, 00-416 Warsaw, Poland; 4 Department of Medical Informatics and Statistics with E-Learning Laboratory, Medical University of Lublin, 20-090 Lublin, Poland; 5 Department of Epidemiology and Health Promotion, School of Public Health, Centre of Postgraduate Medical Education, 00-416 Warsaw, Poland

**Keywords:** self-efficacy, acceptance of illness, health failure, quality of life, AIS, GSES

## Abstract

Health beliefs, disease acceptance, and self-efficacy significantly influence patients’ behavior. This study examined factors associated with self-efficacy and illness acceptance in patients with heart failure (HF). The study, conducted from June 2022 to June 2024, included 231 patients aged ≥ 65 years hospitalized for HF. Self-efficacy and illness acceptance were assessed using the Generalized Self-Efficacy Scale (GSES) and Acceptance of Illness Scale (AIS). The median age of participants was 80 years (72–86); a total of 63.6% were women. The AIS score median was 25, indicating moderate disease acceptance, while the GSES score median was 30, reflecting relatively high self-efficacy. Lower GSES scores were associated with a history of cancer (*p* = 0.002) and geriatric depression (*p* = 0.000). Poor illness acceptance was linked to prior myocardial infarction (*p* = 0.020), atrial fibrillation (*p* = 0.008), stroke (*p* = 0.040), depression (*p* = 0.000), and frailty (*p* = 0.000). Frailty (OR 0.81) and cancer history (OR 3.08) independently predicted self-efficacy, while lower illness acceptance was linked to older age (OR 0.95), stressful events (OR 0.53), stroke (OR 0.26), and improved by physical activity (OR 1.22). Our results indicated that older HF patients exhibit high self-efficacy but moderate illness acceptance. Self-efficacy is influenced by frailty and cancer history, while illness acceptance by age, stress, stroke, and physical activity.

## 1. Introduction

Heart failure (HF) is a chronic, progressive condition characterized by the heart’s inability to pump blood effectively to meet the body’s needs. It affects approximately 65 million people worldwide. In European countries, the incidence of HF ranges from 1% to 4%, but among individuals over the age of 65, the prevalence rises to approximately 12% (Groenewegen et al., 2020). HF leads to a significant decline in daily functioning (Sadeghiazar et al., 2022) and is one of the most common causes of repeated hospitalizations—over half of patients with HF are readmitted within a year of discharge (Schjødt et al., 2017). The burden of HF is increasing due to the aging population, posing a growing challenge to healthcare systems.

In the context of managing HF, patients’ beliefs—particularly their beliefs about their ability to cope with difficult health situations—play a crucial role in engaging in health-related behaviors (Fava et al., 2023). These beliefs may largely determine whether an individual initiates and maintains beneficial lifestyle changes, complies with medical recommendations, and avoids behaviors detrimental to health. Such undesirable behaviors may include physical inactivity, poor dietary adherence, medication noncompliance, or continued smoking and alcohol consumption (Whitehall et al., 2021). Therefore, beliefs that foster active coping and behavioral adaptation can support patients in achieving better health outcomes or even preventing disease progression (T. Wang et al., 2022).

Among these beliefs, one of the most important is self-efficacy—the confidence in one’s ability to take action and persist in health-promoting behaviors, even in the face of adversity. Self-efficacy is not synonymous with locus of control, though the two are correlated. While locus of control refers to a generalized belief about the sources of control over life events (internal vs. external), self-efficacy is a task-specific belief in personal competence to execute actions that produce desired outcomes. In health contexts, higher self-efficacy has been linked with improved adherence to treatment regimens, better management of chronic diseases, increased engagement in physical activity, smoking cessation, and dietary changes. It is also positively associated with mental well-being and quality of life (Remm et al., 2023; Affendi et al., 2018).

Previous research shows that individuals with an internal locus of control—believing that their health depends on their own actions—are more likely to take responsibility for their health and engage in appropriate health behaviors. In contrast, those with low self-efficacy are more likely to feel helpless or resigned, which can lead to disengagement from treatment, passive attitudes, and persistence in harmful habits (Jaarsma et al., 2017; Whitehall et al., 2021). In patients with HF, low self-efficacy has been associated with higher levels of depression and anxiety, poorer adherence to self-care recommendations, and increased hospitalization risk (Luszczynska et al., 2005; Celano et al., 2020; Su et al., 2023).

Similarly, the concept of illness acceptance is critical in understanding patients’ adaptation to chronic disease. Acceptance of illness refers to the process through which an individual adjusts emotionally and behaviorally to the limitations imposed by the disease. Patients who accept their condition are less likely to deny it and more likely to remain active, follow therapeutic recommendations, and experience fewer negative emotional reactions. Research indicates that illness acceptance enhances adherence to medical recommendations, encourages lifestyle modifications, and contributes to better clinical outcomes and health-related quality of life (Mlynarska et al., 2018; Cybulski et al., 2017a; Obiegło et al., 2017). Acceptance is particularly relevant in chronic conditions such as HF, where the trajectory of the disease requires ongoing adjustment.

Despite numerous studies addressing psychological adaptation in chronic diseases, the relationship between general self-efficacy and illness acceptance remains insufficiently explored, particularly among individuals with HF. Moreover, it remains unclear to what extent these psychological constructs are influenced by sociodemographic or clinical characteristics in this patient population. While some studies have examined self-efficacy in HF populations and confirmed its significance (Siennicka et al., 2016; Kärner Köhler et al., 2018), comprehensive analyses of how both self-efficacy and illness acceptance interact and which factors independently influence these variables are still limited.

Therefore, the goals of this study were to assess self-efficacy and illness acceptance, as well as to identify their determinants, in a population of hospitalized patients aged 65 years and older diagnosed with HF.

## 2. Material and Methods

### 2.1. Patients and Setting

Consent to the study was given by the Bioethics Committee at the Centre of Postgraduate Medical Education of Warsaw (resolution No. 73/2022 of 8 June 2022).

We included consecutive patients with health failure hospitalized at the department of internal medicine. The study period is June 2022–June 2024. The criteria for including patients in the study were hospitalization for heart failure and age ≥ 65 years.

### 2.2. Research Tools

#### 2.2.1. Generalized Self-Efficacy Scale (GSES)

The GSES measures the strength of patients’ beliefs about the effectiveness of coping with difficult situations and obstacles. The scale is intended for adults. It consists of 10 statements regarding various personal characteristics of patients, including problem solving, striving for goals and behavior in difficult situations. Patients indicate on a scale from 1 to 4 how much a given statement applies to them (1 means no, 4 means yes). The sum of all points on the scale represents an overall self-efficacy index, which can range from 10 to 40. The higher the score, the greater the self-efficacy. Additionally, the scale scores can be considered as low (10–24 points), medium (25–29 points), and high (30–40 points). The Polish version of the GSES was developed by Juczyński. The reliability of the scale is 0.85 (Cronbach’s alpha coefficient; Juczyński, 2001).

#### 2.2.2. Acceptance of Illness Scale (AIS)

The AIS is intended for adults who are currently ill. The scale contains eight statements describing the negative effects of poor health caused by the disease, including limitations, lack of self-sufficiency, feeling of dependence on others, and reduced self-esteem. Patients indicate the extent to which they agree with a specific statement on a scale from 1 to 5, where 1 means I strongly agree and 5 means I strongly disagree. The patients’ disease acceptance score ranges from 8 to 40 points. The higher the score, the better the acceptance of the disease and the less negative emotions the patient feels in connection with the disease. A low score is considered to be a value below 20 points, an average score is between 20 and 30 points, and a score above 30 points means a high level of acceptance of the disease. The AIS was adapted to Polish conditions by Juczyński. The reliability of the Polish version of the AIS is similar to the original version (Cronbach’s alpha coefficient is 0.82; Juczyński, 2001).

The study also consisted of a personal data sheet, including sociodemographic data, lifestyle data, and health status including comorbidities.

### 2.3. Data Collection

The study participants received the GSES and AIS questionnaires to complete on their own (paper and pencil). Personal identification data, including sociodemographic information, lifestyle, and health status, were collected using the computer-assisted personal interviewing (CAPI) technique.

### 2.4. Statistical Analysis

The statistical analyses have been performed using the statistical suite StatSoft. Inc. STATISTICA (data analysis software system) version 13.0.

The continuous variables were characterized by the arithmetic mean of standard deviation or median (1st–3rd quartile) or max/min (range) and 95% confidence interval. The categorical variables were presented with the use of count and percentage.

In order to check if a quantitative variable derives from a population of normal distribution, the W Shapiro–Wilk test was used. In order to determine dependence, strength, and direction between variables, correlation analysis was used by determining the Pearson and Spearman’s correlation coefficients and using Student’s t-test. Logistic regression analysis was also conducted to identify variables independently associated with disease acceptance. The results were presented as odds ratios (OR) with 95% confidence intervals (CI). In all the calculations the statistical significance level of *p* = 0.05 has been used.

## 3. Results

### 3.1. Characteristics of the Study Group

The data of 231 consecutive hospitalized patients with HF was analyzed. The median age of the patients was 80 (range 72–86) years. Women accounted for 63.6% of the participants (Table 1). The majority of the participants received an average retirement benefit, while only 3% received a very low retirement benefit. A similar percentage of participants lived alone or with another person. The patients experienced 0 to 4 stressful events in the past year, although the majority of them did not experience any or experienced only one. The participants take between 1 and 12 medications daily; the median number of medications taken was 6 (4–7). The majority of patients did not engage in any physical activity.

The predominant type of heart failure was heart failure with preserved ejection fraction (*HFpEF*) with median left ventricular ejection fraction 63 (55–74.5). The majority of patients had hypertension (83.1%). Over 42% of the participants were classified as overweight/obese, and 41.6% had type 2 diabetes. Approximately 11% of all patients had chronic obstructive pulmonary disease (COPD), while nearly 30% had coronary artery disease. Atrial fibrillation was present in 30.1% of the participants. Over 25% of the patients currently have had cancer in the past, over 13% had a past myocardial infraction, and stroke occurred in approximately one in ten of them. Median geriatric depression scoring was in the normal range, with 4 points (2–7), and the Fried frailty index indicated a high level of frailty, with a median 3 points (2–4).

### 3.2. Self-Efficacy and Acceptance of Illness and Its Determinants

Patients with HF had a moderate level of acceptance of the disease: median 25 (18–33) points. The median score of patients obtained on the GSES was 30 (27–35) points.

The analysis revealed an association between GSES score interpretation and cancer diagnosis in the past—individuals with cancer diagnosis in medical history predominantly reported having a higher self-efficacy (*p* = 0.002). The association between depression assessed by geriatric depression scale (GDS) was also found. Patients with a higher level in GDS had a lower self-efficacy (*p* = 0.000). The other variables did not have a statistically significant impact on the level of self-efficacy (*p* > 0.05).

When the factors associated with AIS were analyzed, the association between illness acceptance and cancer diagnosis was not found in the past. The analysis showed that patients with prior myocardial infraction, atrial fibrillation, and stroke in their history had poorer acceptance of their current illness (*p* = 0.020, *p* = 0.008 and *p* = 0.040, respectively). Also, the geriatric depression scale and Fried frailty index were related to lower illness acceptance (*p* = 0.00 for each). Other factors influencing the higher acceptance of illness were a lower number of stressful events during the past year (*p* = 0.014), a higher level of physical activity (*p* = 0.000), and a lower number of taken medications (*p* = 0.05); Table 2.

To explore associations between key psychological and health-related variables, we conducted correlation analyses stratified by sex (Table 3).

Among women, a weak but statistically significant positive correlation was observed between the amount of retirement benefits and illness acceptance (*r* = 0.17, *p* = 0.039). Illness acceptance was also negatively correlated with depressive symptoms, both in men (*r* = −0.42, *p* < 0.001) and women (*r* = −0.35, *p* < 0.001). Additionally, in women only, illness acceptance was significantly associated with self-efficacy (*r* = −0.28, *p* = 0.001), suggesting that lower self-efficacy was related to lower illness acceptance.

In women, self-efficacy was also negatively correlated with obesity (*r* = −0.19, *p* = 0.021), indicating that higher BMI was associated with lower levels of perceived self-efficacy.

Table 4 summarizes the results of logistic regression models identifying factors independently associated with self-efficacy and illness acceptance. Self-efficacy was independently associated with frailty and a history of cancer. Illness acceptance was significantly related to age, stressful life events in the past year, physical activity, and a history of stroke.

## 4. Discussion

Our study showed that patients with HF have a relatively high sense of self-efficacy, which was determined primarily by the presence of cancer diagnosis in medical history. Patients with HF accepted their disease to a moderate extent, while patients diagnosed with frailty and multimorbidity, including myocardial infraction, prior stroke, atrial fibrillation, and depression, scored significantly lower on the AIS. We also found that patients’ physical activity resulted in greater acceptance of the disease. Patients who accepted their illness more highly also had a greater sense of self-efficacy.

General self-efficacy is the belief in one’s own competence to cope with difficult or stressful situations (Luszczynska et al., 2005). Studies in other diseases confirm the relationship between self-efficacy and specific health behaviors (Staszkiewicz et al., 2023); however, there is a lack of research focusing on patients with heart failure (HF). It has been found that self-efficacy affects the regularity of physical exercise, compliance with a diet, quitting smoking, etc., and thus largely determines the achievement of the expected health effects. Moreover, research also confirms the relationship between increased self-efficacy and medication adherence. The consequences of appropriate behaviors may include weight loss and a decrease in BMI, regulation of blood pressure, sugar levels, fitness, exercise capacity, and other indicators that are extremely important in patients with heart failure. Research also emphasizes the role of self-efficacy in maintaining mental health and well-being, including a high ability to adapt to changing conditions related to health and individual physical fitness (Remm et al., 2023). Patients with a greater sense of self-efficacy function better cognitively and experience depression less often (Su et al., 2023).

Haugland et al. (2016) indicate that a GSES score of less than 30 is clinically significant and may influence undesirable health behaviors of patients. Analyzing the elderly population receiving outpatient care, Whitehall et al. (2021) indicated that the average GSES score for this group is 29.34, so it does not differ significantly from the adopted general level of 30 points. Importantly, older people using healthcare services more often achieve a low GSE index than older people not using healthcare services. In this context, hospital care is of the greatest importance, significantly reducing the sense of self-efficacy in patients. However, it has been proven that this result increases as the patient approaches the date of discharge (Volz et al., 2019).

Although no significant differences in self-efficacy in relation to sociodemographic characteristics were observed in our study, Y. Wang et al. (2019) indicate that there may be a gender difference in the GSES results among people aged 60–74. The importance of factors such as age, level of education, income, daily activity, interpersonal relationships, the occurrence of hypertension, mental health, and self-assessment of health were also emphasized. In our study, it was emphasized that in women, but not in men, self-efficacy was correlated with self-assessed general health status. This may indicate that women’s belief in their ability to manage health-related challenges is more strongly influenced by how they perceive their own health. In contrast, men’s self-efficacy appears less dependent on subjective health assessment, suggesting possible gender differences in the psychological mechanisms shaping self-confidence in coping with illness. The relationship between the GSES and self-assessment of health is also emphasized by other authors (Kärner Köhler et al., 2018). A Polish prospective multicenter study using GSES among 758 patients (age: 64 ± 11 years) with systolic HF indicates that self-efficacy was perceived as high (63%) or moderate (27%). There was no relationship between the GSES score and the severity of HF, the duration of HF, the presence of comorbidities, and the treatment used. However, it was emphasized that higher self-efficacy may result in a lower incidence of depression in patients (Siennicka et al., 2016). Similarly, another Polish study, conducted among 300 people >60 years of age, indicated that the respondents had a relatively high (between medium and high) subjective sense of self-efficacy (average of 29 points) (Cybulski et al., 2017b).

Similarly to self-efficacy, acceptance of the disease plays a significant role in patients’ attitudes, subjective quality of life and health outcomes (Mlynarska et al., 2018; Cybulski et al., 2017a; Obiegło et al., 2017). In our study, patients with HF had a moderate level of disease acceptance in the AIS. Interestingly, many other patient groups report a higher acceptance of the disease. For example, elderly people with diabetes, 30% of whom had ischemic heart disease, showed acceptance of the disease on the AIS of 29 points (Bonikowska et al., 2021). In Polish oncological patients, the average score was 27.62 on the AIS (Czerw et al., 2022). Disease acceptance studies conducted among patients with HF, however, show similar results to those in our study. In the study by Sadeghiazar et al. (2022) among 273 hospitalized patients diagnosed with HF, a dominant moderate level of disease acceptance was found (24.9 ± 6.79). Similar results were published among Polish patients with HF; however, in our study, a much more significant percentage of patients indicated low/no acceptance of the disease (over 30%), while in the study by Mlynarska et al. (2018), less than 4.5% of patients with HF had a low level of acceptance of the disease. Obiegło et al. (2017), examining the acceptance of the disease in patients with HF, indicate that older patients achieve worse results on the AIS than younger people. Although some groups of patients reported a relationship between the results on the AIS and sociodemographic variables (professional status, level of education, place of residence, and income) (Czerw et al., 2022), our study highlighted such a relationship only in women for income.

Self-efficacy is often a determinant of patients’ intentions and actions. A sense of high self-efficacy may play an important role in adherence to health behaviors among people with HF, influencing the acceptance of the disease and the subjective, health-related quality of life. Factors independently related to the self-efficacy and the acceptance of illness, that could be modifiable, such as frailty or physical activity, might become a intervention goal. Research conducted among patients with HF indicates a significant role of psychological factors in the disease, as well as the relationship between positive psychological constructs and compliance with physical activity, diet, and medication recommendations. Such reports suggest that among patients with HF, interventions aimed at strengthening positive constructs (including self-efficacy and acceptance of the disease) may be of great importance in achieving the expected health outcomes (Celano et al., 2020).

### Limitations of the Study

The authors are aware of the limitations resulting from the study. The study covers a relatively small number of patients and was conducted in one center. The study group consisted of older people, and thus the sense of self-efficacy or acceptance of the disease may be lower than the average of all heart failure patients. Therefore, the conclusions from our study should not be generalized to all patients with HF.

Second, the study relied on self-reported data, which may be subject to recall bias or social desirability bias. Third, due to the cross-sectional design, causal relationships cannot be inferred. Finally, the number of covariates included in the analysis was limited, raising the possibility that some observed associations may be influenced by unmeasured confounding factors.

## 5. Conclusions

Older, hospitalized patients with HF have a relatively high sense of self-efficacy, but at the same time a moderate level of acceptance of the disease. Age, stressful events, and stroke in the patient’s history, as well as physical activity, are independently associated with the acceptance of illness. Cancer in the history and frailty were independently related to the self-efficacy. Interventions aimed at maintaining or strengthening self-efficacy and the acceptance of health status in this group of patients may be important for effective changes in health behaviors and achieving better clinical effects. Strategies aimed at strengthening patients’ psychological aspects are important to keep patients in good health and physically active for as long as possible.

## Figures and Tables

**Table 1:** The characteristics of the analyzed group.

Variable	Median [1st–3rd Quartile] or Number (%)
Age, years	82 [72–86]
Height, cm	1.63 [1.56–1.70]
Weight, kg	71 [61–83.75]
Sex	
Females	147 (63.6)
Males	84 (36.4)
AIS	25.0 [18.0–33.0]
GSES	31.0 [27.0–35.0]
**Socioeconomic indicators**	
*Retirement benefits*	
Very low	12 (5.2)
Low	45 (19.5)
Average	147 (63.6)
High	27 (11.7)
*Accommodation*	
Alone	108 (46.8)
With company	121 (52.4)
Nursing home	2 (0.9)
*Stressful events last year*	
0	124 (53.7)
1	61 (26.4)
2	31 (13.4)
3	12 (5.2)
4	3 (1.3)
**Medical variables**	
*Self-assessment of general health status*	
Unhealthy	31 (13.4)
Average	114 (49.4)
Healthy	86 (37.2)
Self-reported physical activity, per week	2 [0–7]
Number of medication intake	6 [4–7]
Geriatric depression scale	4 [2–7]
Fried frailty index	3 [2–4]
Left ventricular ejection fraction, %	63.0 [55.0–74.5]
*Laboratory parameters*	
Hemoglobin	12.2 [10.7–13.1]
Creatinine	0.99 [0.8–1.31]
Vitamin D	35.2 [20.5–45.0]
Glucose	101 [93–123]
LDL cholesterol	83.6 [62.5–109.0]
NTproBNP	788.0 [333.6–2884.0]
TSH	1.35 [0.84–2.00]
*Medical history data*	
Obesity	98 (42.4)
Neoplasm in the history	58 (25.1)
Chronic coronary syndrome	68 (29.4)
Prior myocardial infraction	31 (13.4)
COPD	25 (11.3)
Atrial fibrillation	88 (30.1)
Hypertension	192(83.1)
Prior stroke	24 (10.4)
Diabetes	96 (41.6)

AIS—Acceptance of Illness Scale. COPD—chronic obstructive pulmonary disease. GSES—Generalized Self-Efficacy Scale.

**Table 2:** The association between the Acceptance of Illness Scale (AIS), the General Self-Efficacy Scale (GSES), and socioeconomic and clinical variables (Student’s *t*-test).

Variable	AIS < 30 Median [1st–3rd Quartile] or Number (%) n = 149	AIS ≥ 30 Median [1st–3rd Quartile] or Number (%) n = 82	*p*	GSES < 30 Median [1st–3rd Quartile] or Number (%) n = 90	GSES ≥ 30 Median [1st–3rd Quartile] or Number (%) n = 141	*p*
Age, years	83 [76–87]	80 [76–84]	0.004	82 [78–86]	81 [75–85]	0.142
Height, cm	1.62 [1.55–1.70]	1.63 [1.57–1.73]	0.320	1.62 [1.55–1.68]	1.63 [1.57–1.72]	0.147
Weight, kg	71 [60–84]	71 [62–83]	0.972	72 [61–83]	71 [61–84]	0.681
Sex			0.248			0.186
Females	99 (66.44)	48 (58.54)		62 (68.89)	85 (60.28)	
Males	50 (33.56)	34 (39.72)		28 (31.11)	56 (39.72)	
*Socioeconomic indicators*						
*Retirement benefits*			0.084			0.569
Very low	9 (6.04)	3 (3.66)		1 (1.11)	11 (7.80)	
Low	32 (21.48)	13 (15.855)		25 (27.78)	20 (14.18)	
Average	94 (63.09)	53 (64.63)		55 (61.11)	92 (65.25)	
High	14 (9.39)	13 (15.855)		9 (10.00)	18 (12.77)	
*Accommodation*			0.372			0.756
Alone	73 (48.99)	35 (42.68)		40 (44.44)	68 (48.23)	
With company	74 (49.67)	47 (57.32)		50 (55.56)	71 (50.35)	
Nursing home	2 (1.34)	0 (0.00)		0 (0.00)	2 (1.42)	
*Stressful events last year*	1 [0–2]	0.49 [0–1]	0.014	0.00 [0–1]	0 [0–1]	0.963
*Medical history data*						
*Self-assessment of general health status*			0.522			0.088
Unhealthy	25 (16.78)	6 (7.32)		16 (17.78)	15 (10.64)	
Average	68 (45.64)	46 (56.10)		45 (50.00)	69 (48.94)	
Healthy	56 (37.58)	30 (36.58)		29 (32.22)	57 (40.42)	
*Self-reported physical activity, per week*	1 [0–5]	5 [1–7]	0.000	2 [0–5]	3 [0–7]	0.412
*Number of medication intake*	6 [5–7]	5 [3–7]	0.005	6 [4–7]	5 [3–7]	0.764
Geriatric depression scale	5 [3–8]	2 [1–4]	0.000	5 [3–8]	3 [1–5]	0.000
Fried frailty index	3 [3–4]	2 [0–3]	0.000	3 [2–4]	3 [1–4]	0.051
Obesity	64 (42.95)	34 (41.46)	0.730	42 (46.67)	56 (39.72)	0.342
Neoplasm in the history	41 (27.52)	17 (20.73)	0.242	13 (14.44)	45 (31.91)	0.002
Chronic coronary syndrome	46 (30.87)	22 (26.83)	0.495	22 (24.44)	46 (32.62)	0.153
Prior myocardial infraction	26 (17.45)	5 (6.10)	0.020	10 (11.11)	21 (14.89)	0.379
COPD	16 (10.74)	9 (10.98)	0.906	10 (11.11)	15 (10.64)	0.755
Atrial fibrillation	66 (44.30)	22 (26.83)	0.008	34 (37.78)	54 (38.30)	0.839
Hypertension	123 (82.55)	69 (84.15)	0.695	75 (83.33)	117 (82.98)	0.944
Prior stroke	20 (13.42)	4 (4.88)	0.040	8 (8.89)	16 (11.35)	0.517
Diabetes	63 (42.28)	33 (40.24)	0.625	39 (43.33)	57 (40.43)	0.884
*Laboratory parameters*						
Hemoglobin, g/dL	11.9 [10.5–13.0]	12.6 [11.3–14.0]	0.003	12.2 [10.8–13.0]	12.1 [10.6–13.3]	0.788
Creatinine, mg/dL	1.00 [0.82–1.33]	0.97 [0.77–1.30]	0.374	1.00 [0.80–1.28]	0.99 [0.82–1.33]	0.692
Vitamin D, ng/mL	32.70 [18.90–41.90]	38.20 [25.37–50.32]	0.099	36.90 [17.40–45.90]	33.45 [21.50–44.18]	0.852
Glucose, mg/dL	102.5 [93.5–127.0]	100.0 [93.0–113.0]	0.354	100.0 [92.0–116.0]	102.0 [94.0–128.0]	0.177
LDL cholesterol, mg/dL	81.8 [60.4–108.2]	87.9 [67.8–110.8]	0.537	90.0 [63.8–109.0]	80.1 [60.8–109.0]	0.521
NTproBNP, pg/mL	966 [416–4112]	477 [194–2239]	0.005	696 [299–3453]	915 [348–2717]	0.513
TSH, µL/mL	1.33 [0.84–2.12]	1.35 [0.78–1.84]	0.785	1.38 [0.92–2.25]	1.31 [0.75–1.82]	0.130

**Table 3:** Variables independently related to the General Self-Efficacy Scale and the Acceptance of Illness Scale.

Variable	GSES OR [Cl 95%]	AIS OR [Cl 95%]
Fried frailty index	0.81 [0.67–0.99]	-
Neoplasm in the medical history	3.09 [1.52–6.28]	-
Age	0.98 [0.86–1.12]	0.95 [0.91–0.99]
Stroke in the history	-	0.26 [0.08–0.84]
Physical activity	-	1.22 [1.11–1.35]
Stressful events last year	-	0.53 [0.37–0.76]

**Table 4:** Correlations between socioeconomic and clinical variables and the Acceptance of Illness Scale (AIS) and the General Self-Efficacy Scale (GSES), considering sex.

Variable	AIS R Spearman				GSES R Spearman			
	M	*p* Value	F	*p* Value	M	*p* Value	F	*p* Value
**Socioeconomic indicators**								
Retirement benefits	0.01	0.929	0.17	0.039	−0.03	0.783	0.12	0.149
Accommodation	0.13	0.232	−0.05	0.586	−0.03	0.795	0.09	0.290
Stressful events last year	−0.11	0.321	−0.25	0.002	−0.06	0.607	−0.06	0.505
Geriatric depression scale	−0.42	0.000	−0.35	0.000	−0.05	0.662	−0.28	0.001
**Medical variables**								
Self-assessment of general health status	0.19	0.078	0.12	0.137	0.01	0.936	0.29	0.000
Self-reported physical activity, per week	0.33	0.004	0.18	0.032	0.18	0.127	0.05	0.519
Number of medication intake	−0.12	0.268	−0.21	0.011	0.21	0.059	−0.13	0.126
Frailty index	−0.38	0.001	−0.45	0.000	−0.24	0.035	−0.14	0.092
**Medical history data**								
Obesity	−0.07	0.513	0.06	0.486	0.20	0.066	−0.19	0.021
Neoplasm in the history	−0.07	0.552	−0.14	0.085	0.09	0.404	0.15	0.070
Chronic coronary syndrome	0.02	0.850	−0.12	0.154	0.05	0.667	0.12	0.155
Prior myocardial infraction	−0.14	0.192	−0.17	0.039	0.02	0.876	0.07	0.375
COPD	−0.09	0.431	−0.02	0.835	−0.16	0.157	0.11	0.181
Atrial fibrillation	−0.10	0.381	−0.19	0.023	−0.13	0.238	−0.02	0.767
Hypertension	0.08	0.474	0.02	0.845	0.21	0.057	−0.10	0.215
Prior stroke	−0.13	0.237	−0.05	0.550	0.02	0.850	0.05	0.517
Diabetes	0.00	0.968	0.01	0.916	0.11	0.341	−0.04	0.644

## Data Availability

Data are contained within the article.

## Abbreviations

AF	atrial fibrillation
AIS	Acceptance of Illness Scale
CAD	coronary artery disease
CAPI	computer-assisted personal interviewing
COPD	chronic obstructive pulmonary disease
GSES	Generalized Self-Efficacy Scale
HF	heart failure
HFmEF	heart failure with mid-range ejection fraction
HFpEF	heart failure with preserved ejection fraction
HFrEF	heart failure with reduced ejection fraction
NYHA	New York Heart Association
SD	standard deviation
